# RNA-seq analysis identifies an intricate regulatory network controlling cluster root development in white lupin

**DOI:** 10.1186/1471-2164-15-230

**Published:** 2014-03-25

**Authors:** David Secco, Huixia Shou, James Whelan, Oliver Berkowitz

**Affiliations:** 1 Australian Research Council Centre of Excellence in Plant Energy Biology, University of Western Australia, Crawley, WA 6009, Australia; 2 State Key Laboratory of Plant Physiology and Biochemistry, College of Life Sciences, Zhejiang University, Hangzhou 310058, China; 3 Joint Research Laboratory in Genomics and Nutriomics, College of Life Sciences, Zhejiang University, Hangzhou 310058, China; 4 Department of Botany, School of Life Science, La Trobe University, Bundoora, Victoria 3086, Australia; 5 School of Plant Biology, University of Western Australia, Crawley, WA 6009, Australia

**Keywords:** Cluster root, White lupin, Root development, Phosphate, RNA-seq, *de novo* transcriptome

## Abstract

**Background:**

Highly adapted plant species are able to alter their root architecture to improve nutrient uptake and thrive in environments with limited nutrient supply. Cluster roots (CRs) are specialised structures of dense lateral roots formed by several plant species for the effective mining of nutrient rich soil patches through a combination of increased surface area and exudation of carboxylates. White lupin is becoming a model-species allowing for the discovery of gene networks involved in CR development. A greater understanding of the underlying molecular mechanisms driving these developmental processes is important for the generation of smarter plants for a world with diminishing resources to improve food security.

**Results:**

RNA-seq analyses for three developmental stages of the CR formed under phosphorus-limited conditions and two of non-cluster roots have been performed for white lupin. In total 133,045,174 high-quality paired-end reads were used for a *de novo* assembly of the root transcriptome and merged with LAGI01 (*Lupinus albus* gene index) to generate an improved LAGI02 with 65,097 functionally annotated contigs. This was followed by comparative gene expression analysis. We show marked differences in the transcriptional response across the various cluster root stages to adjust to phosphate limitation by increasing uptake capacity and adjusting metabolic pathways. Several transcription factors such as PLT, SCR, PHB, PHV or AUX/IAA with a known role in the control of meristem activity and developmental processes show an increased expression in the tip of the CR. Genes involved in hormonal responses (*PIN, LAX, YUC*) and cell cycle control (*CYCA/B, CDK*) are also differentially expressed. In addition, we identify primary transcripts of miRNAs with established function in the root meristem.

**Conclusions:**

Our gene expression analysis shows an intricate network of transcription factors and plant hormones controlling CR initiation and formation. In addition, functional differences between the different CR developmental stages in the acclimation to phosphorus starvation have been identified.

## Background

Phosphorus (P) is often one of the most limiting plant nutrients in soils leading to impeded plant growth and development. Although inorganic phosphate (Pi) is often abundant in soils, availability to the plant is limited by slow diffusion and fixation, e.g. as aluminum or iron oxide complexes [1]. Therefore fertilisation with Pi derived from mined rock phosphate is essential in high intensity agriculture to maintain crop productivity. However, Pi is a non-renewable resource and, in conjunction with diminishing energy sources, this will in the future impact on fertiliser costs and food security [2]. It is therefore of high importance to fully understand the mechanisms employed by plants under P deficiency as a knowledge base for the development of smart plants with increased capacity to mine and efficiently use Pi. This will become an increasingly important factor in the necessary sustainable intensification of productivity to feed a growing world population.

Plants have developed adaptive strategies to cope with limiting Pi availability. These physiological and developmental adaptations are collectively referred to as phosphate starvation response (PSR). This includes the transcriptional induction of genes coding for high-affinity phosphate transporters (PHTs) or purple acid phosphatases (PAPs) to increase uptake capacity and mobilisation of Pi [3]. In addition, members of the SPX domain containing protein family (e.g. PHO1, SPX-MFS, NLA) involved in the regulation of Pi homeostasis, and enzymes re-modelling membrane lipid composition to remobilise Pi from phospholipids such as monogalactosyl diacylglycerol synthases (MGDs) and UDP-sulfoquinovose:DAG sulfoquinovosyltransferase (SQD2), are also differentially expressed [4,5]. A central component of the PSR is the miRNA399/PHO2/PHR1 regulon. In Pi limited conditions the expression of miRNA399 is highly up-regulated by the transcription factor PHR1 leading to the degradation of its target PHO2. This E2 ubiquitin conjugase regulates protein turn-over of target proteins that include PHO1 and Pi transporters [6-9]. Morphological changes in root system architecture such as a decreased primary root growth and increased lateral root formation have been analysed on the molecular level in Arabidopsis [10]. Specialised root structures termed cluster roots formed on lateral roots by dense rootlets of limited growth can be found in a diverse range of plant families such as the Fabaceae and Proteaceae [11,12].

White lupin (*Lupinus albus*) has become a model plant for the analysis of cluster root (CR) biology [13]. CR formation is a highly complex process in which developmental changes such as the initiation of lateral roots in addition to metabolic adjustments e.g. increased carboxylate production and secretion, have to be integrated to ensure their functioning. In white lupin foliar application of Pi inhibits cluster root development, providing evidence that systemic signalling is involved in cluster root formation [14]. By contrast, local sensing of Pi-rich soil patches induces cluster root formation to increase root surface area [15]. In addition, the secretion of large amounts of carboxylates such as citrate and malate from mature cluster roots (‘exudative burst’) generates a high and localised concentration able to mobilise Pi from insoluble forms [16].

The plant hormones auxin and cytokinin are important players in the regulation of cluster root development which is not surprising given their central function in the control of root development identified in non-cluster root forming model species such as Arabidopsis [17]. In this species auxin is involved in the root architectural changes under Pi deficiency leading to reduced primary root growth and increased lateral root formation [18-20]. Cytokinin has an antagonistic role to auxin by promoting cell differentiation, inhibition of lateral root formation and preventing their formation in close proximity to each other [21-23]. In white lupin there is also increasing evidence for an involvement of auxin in the control of cluster root formation. The exogenous application of auxin promotes cluster root formation while auxin transport inhibitors have an impeding effect. Also, several genes involved in auxin signalling have been identified as expressed in developing cluster roots [24-26]. Similarly to observations on lateral root initiation in Arabidopsis, cytokinin also inhibits the formation of cluster roots in white lupin under Pi deficiency [26,27].

Here we analyse cluster root formation in white lupin under phosphate limitation by RNA-seq. This is the first study that analyses different developmental stages of a forming cluster root on a transcriptomic scale. Our next-generation sequencing data contribute to the available information on the white lupin transcriptome as a further step in developing this species into a model plant for cluster root biology. We identify a complex network of hormone pathways and transcription factors involved in the regulation of early cluster root formation. In addition, functional differences between the various parts of the cluster root in the acclimation to phosphate deficiency are discovered.

## Results

### Assembly of RNA-seq data and integration into LAGI01

For the analysis of gene expression in the developing white lupin roots, tissue from root tips (TCR), immature (ICR) and mature cluster roots (MCR) that developed in plants grown under P-deficient as well as root tips (TR) and mature roots (MR) of plants grown under P-sufficient conditions were harvested at the 6- to 8-trifoliolate stage. The generated RNA-seq libraries of three independent biological replicates for each treatment were then paired–end sequenced. After filtering for reads with a Phred score of +30 and a length above 90 bp a total of 133,045,174 high-quality reads were used for a *de novo* assembly of the white lupin root transcriptome. Using the Velvet/Oases pipeline [28] a total of 46,383 contigs with an average size of 896 bp were obtained. The longest contig (LAGI02_264) is a 29,640 bp fragment that originated from contaminating mitochondrial DNA while the longest protein coding contig (LAGI02_265) is 16,579 bp in size with 79% identity to the *Cicer arietinum* MIDAS-like mRNA, highlighting the ability of our assembly parameters to generate an assembly with high contig quality and lengths. In order to further improve the quality of our assembly, we merged it with a recently published white lupin transcriptome assembly (LAGI01, [26]), thus generating LAGI02 (Additional files 1, 2 and 3). After removal of contigs with a length below 200 bp, LAGI02 contains 65,097 contigs with an average size of 1,625 bp and a total length of 105,789,289 bp (Table 1, Additional file 4: Figure A). For the functional annotation contigs were first assigned to the best BLASTX hit against the NCBI database with a cut-off of E < 10^−15^. The majority of primary BLAST hits are against sequences from legume species (Additional file 4: Figure B). Targeted homology searches against the *Glycine max* (Gmax_189 release) and Arabidopsis (TAIR10 release) genomes using BLAT were able to assign putative white lupin homologs for 96% and 44%, respectively (Additional file 1). This extensive homology of soybean and white lupin sequences was then used to assign gene ontology terms to the LAGI02 contigs. Comparison of GOslim terms in the three main GO classes biological process (48% of all assigned GO terms), cellular component (28%) and molecular function (24%) showed no major difference in the GOslim term distributions for the two species in accordance with their close phylogenetic relationship (Additional file 5). Functional characteristics annotation was also performed according to the Mapman functional bin classification [29], allowing the usage of this versatile tool in the visualisation of gene expression data in white lupin. Again, there were no major differences in the distribution of genes across the functional bins for LAGI02 when compared to the Gmax_189 and TAIR10 genome releases (Additional file 6). Taken together, LAGI02 presents a highly valuable resource for functional genomics studies in this emerging model species, e.g. for the analysis of cluster root development.

**Table 1:** **Characteristics of white lupin *de novo *** **assemblies**

**Parameter**	LAGI01*	**This work**^**^**^	**LAGI02**^**#**^
**Kmer length**	29	67	-
**No. of reads**	277,224,180	133,045,174	-
**No. of contigs**	125,821	46,383	65,097
**Average contig length**	1,155	896	1,625
**% GC**	39.6	40.03	39.4
**Longest contig**	15,514	28,229	26,640
**Total bases**	145,286,000	41,550,474	105,789,289

*reference: [26].

^^^*de novo* assembly of the five analysed root tissues.

^#^merged assemblies after removal of reads < 200 bp and with no BLAST hit.

### P-deficiency induces genes of the classical phosphate starvation response in cluster root tissues

**Figure 1:**
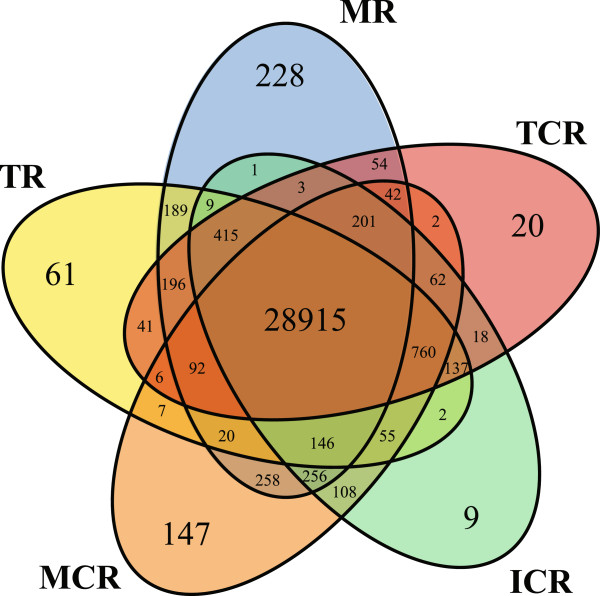
**Tissue-specific expression of LAGI02 contigs at different developmental stages of white lupin roots.** In total 32,204 contigs were expressed above a threshold of FPKM > 5 in at least one of the white lupin root samples. Of these 28,915 were expressed across all tissues while 465 contigs were preferentially expressed in one tissue with a cut-off of a 5-fold higher expression than in all other tissues. For both the cluster (−P) and non-cluster (+P) root the mature part had the highest number of preferentially expressed genes. The ICR had the lowest number, reflecting its intermediary status in cluster root development. Abbreviations: TR, root tip; MR: mature root; TCR: tip of cluster root; ICR, immature cluster root; MCR: mature cluster root.

Phosphorus-deficiency is the main cue to induce cluster root formation in white lupin and hence was used to determine differences in gene expression in the different stages of developing cluster roots (Additional file 7). The RNA-seq data were first used to assess differences in gene expression in the five collected tissues under Pi-sufficient (+P) and Pi-deficient (−P) conditions (Additional file 8). Only transcripts with a FPKM value of above 5 in at least one tissue sample were called as expressed, leaving 32,204 transcripts for further analyses (Figure 1). Of these, 28,915 (90%) transcripts were expressed in all five samples, while the number of genes preferentially expressed in only one sample ranged from 228 transcripts in the MR sample to only 9 transcripts in the ICR sample (Figure 1).

**Figure 2:**
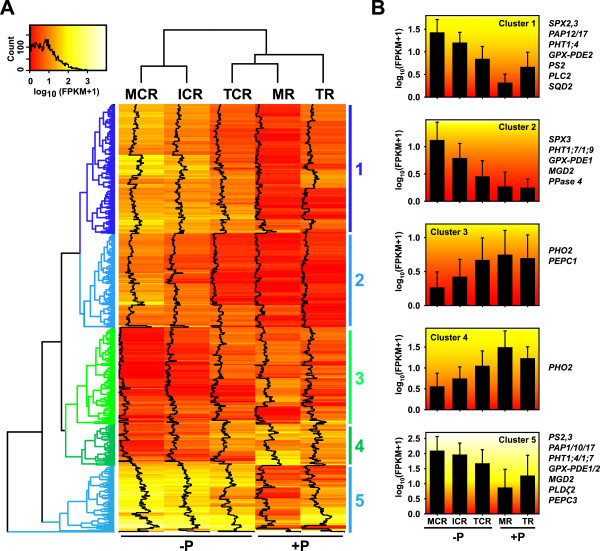
**Hierarchical clustering of LAGI02 contigs differentially expressed under phosphate deficiency. A)** Heatmap of log_10_(FPKM + 1) values for the expression of LAGI02 contigs in the different analysed white lupin root tissues. Shown are only the 835 contigs with an higher than 8-fold up- or down-regulation in at least one pair-wise comparison of tissues. Hierarchical clustering using the Manhattan distance identified five main clusters. Clusters 1, 2 and 5 contained genes up-regulated by P-deficiency and more highly expressed in the mature cluster root than in the immature cluster root and the tip. **B)** Average expression pattern of genes in the main five clusters (means ± SD). In addition, known P-deficiency responsive genes within each cluster are given. For contigs the name of the identified homologous Arabidopsis genes are given. Abbreviation: *SPX*, *SPX DOMAIN; PAP*, *PURPLE ACID PHOSPHATASE*, *PHT1, PHOSPHATE TRANSPORTER1*; *GPX-PDE, GLYCEROPHOSPHODIESTER PHOSPHODIESTERASE; PS, PHOSPHATE STARVATION*, *PLC, PHOSPHOLIPASE C; SQD, SULFOQUINOVOSYLDIACYLGLYCEROL; MGD, MONOGALACTOSYL DIACYLGLYCEROL SYNTHASE; PPase*, *PHOSPHORYLASE*, *PHO2*, *PHOSPHATE2*; *PEPC, PHOPSPHOENOLPYRUVATE CARBOXYLASE; PLDζ2, PHOSPHOLIPASEζ2*.

In total 835 (2.5%) transcripts were differentially expressed by more than 8-fold in at least one pairwise comparison of samples form Pi-deficient and Pi-sufficient treatments. Hierarchical clustering of these transcripts identified five main clusters (Figure 2A). Interestingly, while the MCR and ICR group together, the TCR response was more closely related to those of the MR and TR. This indicates that there was a more limited response to P deficiency in the TCR than in the MCR or ICR. Clusters 1, 2 and 5 are composed of transcripts with increased expression under –P condition. These three clusters included many transcripts homologous to the well-established phosphate-starvation induced (PSI) genes coding for e.g. purple acid phosphatases (PAPs), phosphate transporters (PHTs) or SPX proteins, with expression of these genes under –P highest in the MCR (Figure 2B and Additional file 9). The relative response of genes in Clusters 1 and 5 to –P conditions was identical but with genes in Cluster 5 generally having an approximately 3-fold higher expression level. In both these clusters expression of genes was lower in the MR than in the TR tissue while in Cluster 2 these were overall very similar. Clusters 3 and 4 contained transcripts down-regulated by P deficiency including *PHOSPHATE 2* (*PHO2*) and a phosphoenolpyruvate carboxylase (PEPC) isoform as known phosphate starvation-responsive genes (Figure 2B). Our results therefore confirm the transcriptional regulation of gene expression induced by phosphate starvation and the value of LAGI02 as a resource for transcriptomic studies.

### Transcriptional control of metabolic acclimation to P deficiency

Thus far, no genome-wide transcriptomic study investigating the response of different developmental stages of a cluster root has been published. We therefore analysed our dataset for differences in the response of the three tissues under phosphate starvation. When analysing expression of classical PSI genes in the same tissues of plants grown under –P and + P conditions, i.e. MCR vs. MR and TCR vs. TR, genes belonging e.g. to the *PHT1*, *SPX* and *PAP* family showed a very similar response in the two tissue comparisons (Additional file 9). This indicates that the primary transcriptional response of genes directly involved in phosphate acquisition and homeostasis are regulated to a similar degree across the different stages of cluster root development.

**Figure 3:**
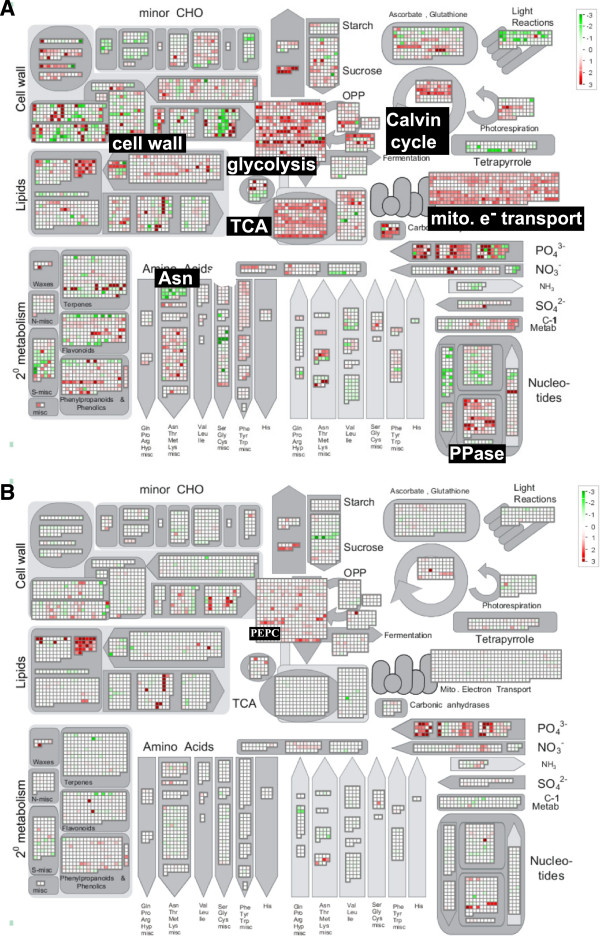
**Mapman representation of differential gene expression for the mature root and root tips under altered P supply.** Comparison of gene expression in the mature root cluster root and non-cluster root **(A, MCR vs. MR)** and root tips **(B, TCR vs TR)**, i.e. P-deficient versus P-sufficient tissues for genes involved in primary metabolism. In the comparison of P/+P mature root many genes related to the TCA cycle, glycolysis or Calvin cycle showed higher expression in the MCR than in the MR while such a response was not observed for the root tips. Here, with the exception of *PEPC*, only genes directly involved in P homeostasis or lipid remodelling showed higher expression in the TCR. Given are log_2_ values for corresponding FPKM ratios. Red colour highlights up-regulated, green colour down-regulated genes.

However, there was a pronounced difference in the expression of genes implicated in the metabolic acclimation to low P supply. In the mature part of the root, phosphate deficiency induced a high number of genes by more than 4-fold coding for enzymes involved in primary carbon metabolism, i.e. the TCA cycle, glycolysis, Calvin cycle, and mitochondrial ATP synthesis (Figure 3A). For the TCA cycle, up-regulated genes code for dihydrolipoamide S-acetyltransferase, malate dehydrogenase and pyruvate dehydrogenase. Among the highest up-regulated glycolysis-related genes are phosphoenolpyruvate carboxylase, glyceraldehyde-3-phosphate dehydrogenase and enolase. Induced Calvin cycle genes are encoding transketolase and aldolase. Most highly regulated among all was malate synthase with a more than 100-fold higher expression in the MCR than in the MR. This was accompanied by an induction of plasma membrane H^+^-ATPases and membrane transporters of the MATE family by about 10-fold (Additional file 10). A similar response was not induced in the root tip of P-deficient plants when compared to the TR in non-cluster roots. The only gene up-regulated to similar degree as in the MCR/MR comparison was PEP carboxylase (Figure 3B).

In addition, several genes coding for membrane transporters were differentially expressed between MCR and TCR (Additional file 10). Interestingly, there was also a differential response of nitrate, ammonium and sulfate transporters. While a nitrate transporter homologous to *NRT1.5* involved in root to shoot transport of NO_3_^−^ in Arabidopsis was more highly expressed in the MCR, several others (e.g. *NRT1.7, NRT2.4*) showed higher expression in the TCR. Similarly, lupin homologs of sulfate transporters belonging to the SULTR3 family (SULTR3;1, SULTR3;5) were more abundant in the MCR and high-affinity transporters such as SULTR1;1 and SULTR1;3 showing higher expression in the TCR (Additional file 10).

Taken together, these results show that with the exception of genes directly involved in uptake and homeostasis of phosphate the cluster root tip was not involved in the metabolic acclimation of the cluster root to phosphate deficiency. Only genes directly involved in the uptake or recycling of phosphate were transcriptionally responding in this tissue. By contrast, the mature part of the cluster root was solely responsible to sustain the exudative burst and necessary anaplerotic reactions, thus also supporting Pi uptake of the root tip by making insoluble Pi available through exudation of carboxylates.

### Regulation of cluster root formation by hormones and transcription factors

**Figure 4:**
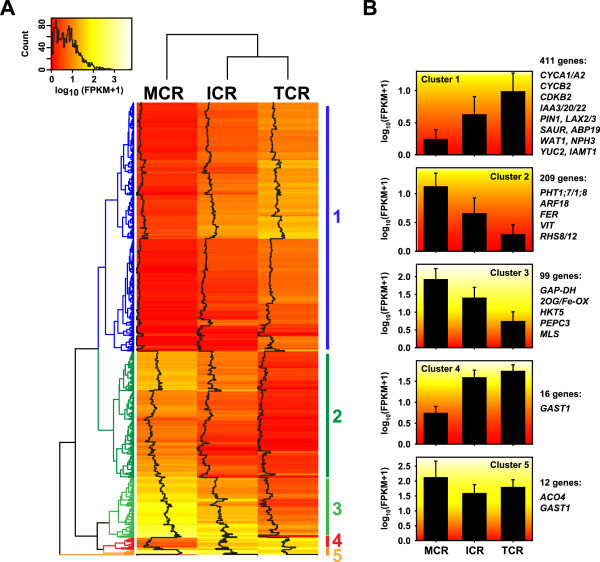
**Hierarchical clustering of genes differentially expressed across developmental stages of cluster root. A)** Hierarchical cluster analysis gave five main clusters for the 747 contigs showing differential expression in at least one pair-wise comparison of the three analysed cluster root tissues. **B)** Average expression pattern of contigs in the main five clusters (Given are means ± SD). Known P-deficiency responsive genes within each cluster are also indicated. Cluster 1 contained genes with highest expression in the root tip of the cluster root and decreasing towards the mature part. GO term analysis identified that this cluster was enriched for genes with functions in cell cycle regulation and auxin-mediated growth control. By contrast, genes in Cluster 2 and 3 showed the opposite expression pattern and are involved in iron homeostasis and oxidative stress response. In comparison, Clusters 4 and 5 only had few genes without any GO term enrichment. For contigs the name of the identified homologous Arabidopsis genes are given. Gene abbreviations are: *2OG/Fe-OX, 2-OXOGLUTARATE AND FE(II)-DEPENDENT OXYGENASE; ABP, AUXIN BINDING PROTEIN; ACO, 1-AMINOCYCLOPROPANE-1-CARBOXYLATE OXIDASE; ARF, AUXIN RESPONSE FACTOR; CYC, CYCLIN; CDK, CYCLIN-DEPENDENT KINASE; FER, GAP-DH, GLYCEROALDEHYDE DEHYDROGENASE; GAST, GIBERELLIC ACID STIMULATED TRANSCRIPT; HKT, HIGH AFFINITY K*^*+ *^*TRANSPORTER; IAA, INDOLE ACETIC ACID-INDUCIBLE; IAMT, IAA CARBOXYLMETHYLTRANSFERASE; LAX, LIKE AUX1; MLS, MALATE SYNTHASE; NPH, NON PHOTOTROPIC HYPOCOTYL; PEPC, PHOSPHOENOL PYRUVATE CARBOXYLASE; PHT, PHOSPHATE TRANSPORTER; PIN, PIN-FORMED; RHS, ROOT HAIR SPECIFIC; SAUR, SMALL AUXIN UP-REGULATED RNA; VIT, VACUOLAR IRON TRANSPORTER; WAT, WALLS ARE THIN; YUC, YUCCA*.

To gain a better understanding of the regulatory processes involved in cluster root formation we next analysed our data for difference in gene expression across the different stages of the cluster root (Figure 4). Our analyses identified 747 genes differentially expressed by more than 8-fold in at least one pair-wise comparison of the three analysed developmental stages. Hierarchical cluster analysis showed five main clusters with Clusters 1 and 2 containing the majority of genes (Figure 4A). Cluster 1 is composed of genes with a low expression in the MCR and increasing expression in the two younger cluster root stages ICR and TCR, respectively (Figure 4B). By contrast, genes in Cluster 2 have a high expression in the MCR, decreasing towards the TCR. Genes in Cluster 3 have an identical pattern across the tissues as Cluster 2 but at a higher expression level. Cluster 4 and 5 contain only a small number of genes compared with the other clusters, with Cluster 4 including genes preferentially expressed in the ICR and TCR, and Cluster 5 showing genes with high expression especially in the MCR (Figure 4B).

**Figure 5:**
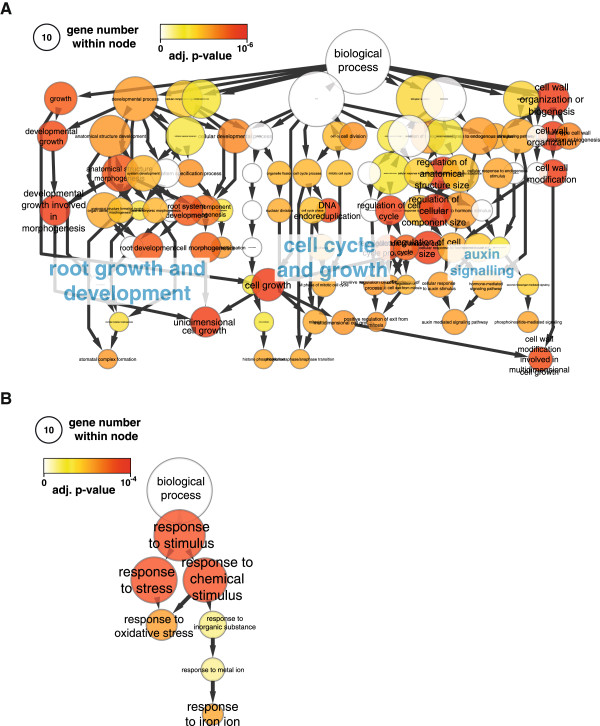
**Analysis of GO term enrichment for differentially expressed genes in the three analysed stages of cluster root development.** Over-represented GO terms for genes with higher expression in **A)** the TCR (see Figure 4, Cluster 1) and **B)** the MCR (see Figure 4, Clusters 2 and 3). Analysis was performed using the BiNGO plugin for Cytoscape with Benjamini-Hochberg FDR correction (p < 0.01) and results plotted in a y-hierarchical representation. The circle areas represent the number of genes associated to a given GO term and color reflects the adjusted p-value.

**Figure 6:**
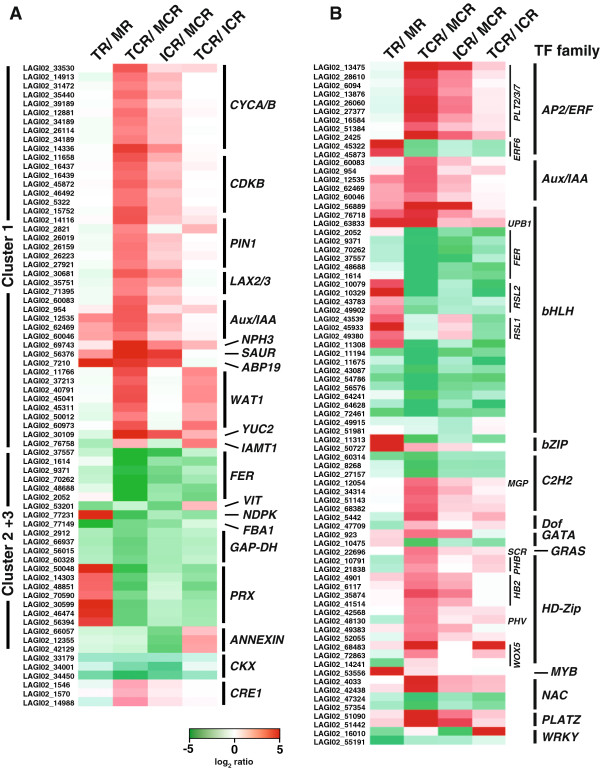
**Differential expression of gene identified by GO term enrichment analysis and of transcription factors. A)** Shown is differential expression of genes associated to enriched GO terms in Cluster 1 (cell cycle, auxin, root development) and Clusters 2 + 3 (oxidative stress, response to iron) of the hierarchical cluster analysis (Figure 4). Additionally, white lupin homologs for two genes involved in cytokinin signalling, cytokinin oxidase (CKX) and the cytokinin receptor CRE1 are presented. For contigs the name of the identified homologous Arabidopsis genes are given. Gene abbreviations are: *FBA, FRUCTOSE 1,6 BIPHOSPHATE ALDOLASE*; *NDPK,NUCLEOSIDE DIPHOSPHATE KINASE; PRX, PEROXIDASE.* For other gene abbreviations see Figure 4. **B)** Differentially expressed transcription factors shown as pair-wise comparisons for non-cluster root and cluster root tissues, respectively. Individual genes were grouped into the corresponding TF families. Gene abbreviations: *ERF, ETHYLENE RESPONSE FACTOR; HB2, HOMEOBOX PROTEIN2*; *MGP, MAGPIE; PHB, PHABULOSA; PHV, PHAVOLUTA; PLT, PLETHORA; RSL, ROOT HAIR DEFECTIVE SIX-LIKE; SCR, SCARECROW; UPB1, UPBEAT1; WOX, WUSCHEL-RELATED HOMEOBOX.* Differential expression of genes in the tissues of the P-sufficient root and P-deficient cluster root tissues in **A)** and **B)** are given as log_2_ of the FPKM ratios.

We next performed analyses for GO term enrichment of genes in Cluster 1 and genes in Clusters 2 and 3 combined. This showed an enrichment of GO terms related to root growth and development, cell cycle/growth and auxin for Cluster 1 (Figure 5A and Additional file 11). Genes associated to these are homologs of cyclins (*CYCA1/2*, *CYCB2*), cyclin-dependent kinases (*CDKB2*), auxin transporters (*PIN1*, *LAX2/3*), auxin signalling-components (*Aux/IAA*, *ABP19*), auxin-dependent cell growth regulators (*NPH3, WAT1*) and auxin-synthesis enzymes (*YUC2, IAMT1*) (Figure 4B). Most of these genes show higher expression in the younger stages of the cluster root than in the mature part and little difference in a TCR vs. ICR comparison (Figure 6A). Only the *WAT1* gene, coding for a tonoplast-localised protein involved in secondary wall formation, showed a higher expression in the TCR than in the ICR and the MCR. In the P-sufficient white lupin root a similar expression pattern of these genes was absent, likely because an initiation and formation of laterals to form a cluster root was not induced.

For Clusters 2 and 3 we identified only a limited number of GO terms enriched (Figure 5B and Additional file 11) with associated genes relating to oxidative stress (*PRX, FBA, GAP-DH, ANNEXIN*) and iron homeostasis (*FER, VIT*) (Figure 6A). These genes have lower expression in the younger stages of the cluster roots, e.g. homologs of the iron uptake regulator FER and peroxidase show a 30-fold and 15-fold lower expression in the TCR than the MCR (Figure 6A). This is in agreement with results identifying oxidative stress through reactive oxygen (ROS) production as a confounding effect of phosphate starvation [30,31]. Our results provide evidence that this is more so in the mature part of the cluster root, likely as a consequence of the higher metabolic activity. However, the peroxidase homologs were more highly expressed in the root tip than in the mature part of P-sufficient plants.

Given the extensive differences observed in the transcriptomes of the three cluster root stages and the complex developmental changes involved it is not surprising we also observed differences in gene expression levels for several transcription factors (TFs) across the three cluster root samples other than the already described *AUX*/*IAA* and *FER*. For several contigs with homology to the AP2/ERF class TFs of the PLETHORA family (PLT2/3/7) expression was about 30- and 10-fold higher in the cluster root tip than in the immature and mature cluster root tissues, respectively, while it was not changed in the P-sufficient MR and TR (Figure 6B). Similarly, homologs of the homeodomain-leucin zipper transcription factor ATHB2 were most highly expressed in the TCR. Several other TFs (SCR, MGP, WOX5, PHB, PHV) of the regulatory network controlling the activity and cell fate within the root meristem also show higher expression in the TCR (Figure 6B). For these there is an at least 8-fold higher expression in the TCR than in the MCR and little difference between TR and MR. Interestingly, the expression of a homolog of the bHLH transcription factor UPB1 (UPBEAT1), a repressor of peroxidase expression and central regulator of ROS balance controlling the transition from proliferation to differentiation in the root [32], was contrasting with peroxidase expression in the cluster roots but not the non-cluster root (Figure 6A and B).

Cytokinin (CK) is also an important regulator of cell differentiation within the root and also repressed the expression of *PHB* and *miRNA165*. Therefore CK is also a component of the regulatory network governing root development [23]. Correspondingly, several LAGI02 contigs with sequence homology to the Arabidopsis cytokinin oxidases (CKX) involved in CK degradation, are down-regulated by about 5-fold in the TCR when compared to the MCR (Figure 6A). At the same time a lupin homolog of the Arabidopsis cytokinin receptor CRE1 was about 5-fold more highly expressed in the TCR than in the MCR. These gene expression patterns suggest an increase in local cytokinin concentration and CK sensitivity towards the root tip to remove inhibition of lateral root development and observed responsiveness of PSR genes [33,34].

Thus, our data identified a complex regulatory circuit of transcription factors and of the plant hormones auxin and cytokinin involved in the initiation of cluster roots in white lupin. These prime lateral root formation, promote their initiation and elongation under P-limited conditions.

### Identification of pri-miRNAs expressed in the cluster root

**Figure 7:**
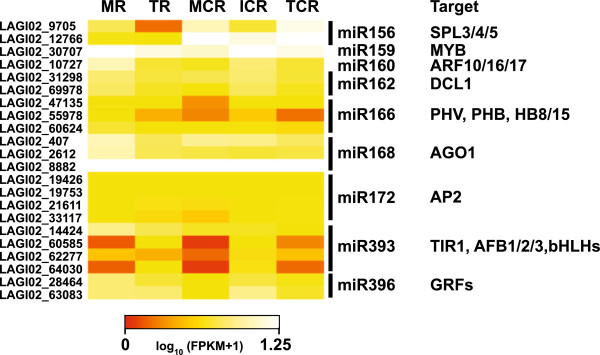
**Heatmap for pri-miRNA transcript abundance in the five analysed root tissues.** Transcript abundance of the identified pri-miRNAs was similar across the analysed root tissues of P-deficient and P-sufficient plants with the exception of pri-miRNA 166 and 393. miRNA156 is 2 to 3-fold more abundant in tissues from P-deficient cluster roots. The pri-miRNA166 is less abundant in the MCR and TCR while pri-miRNA393 was more abundant in the TR and ICR. Although the detection of these pri-miRNAs was indication of their functional role in the regulation, their abundance was not a reliable measure for post-transcriptional regulation of their turn-over and thus degradation of their downstream targets. Targets of the miRNAs are involved in auxin signalling (AFB, AUXIN SIGNALING F-BOX; ARF, AUXIN RESPONSE FACTOR; TIR1, TRANSPORT INHIBITOR RESPONSE1), development (HB2, HOMEOBOX PROTEIN; GRF, GROWTH REGULATING FACTOR; PHB, PHABULOSA; PHV, PHAVOLUTA; SPL, SQUAMOSA PROMOTER BINDING-LIKE) or miRNA processing (AGO1, ARGONAUTE1; DCL1, DICER LIKE1).

LAGI02 contains sequences with homology to known primary transcripts of miRNAs (pri-miRNAs) and allowed us the identification of corresponding pre-miRNA hairpin structures and their mature miRNA sequence. These were very similar between lupin and soybean with minor difference in the hairpin structure outside of the highly conserved miRNA/miRNA* region (Additional file 12). Recent studies have profiled the abundance of miRNAs and identified putative targets of lupin miRNAs [26,35]. Our data add to the complement of miRNA in the developing cluster roots. Interestingly, we did not identify a precursor of miRNA399, a key regulator of the phosphate starvation response [6,7], in our dataset although lupin homologs of its target *PHO2* contain a sequence complementary to miRNA399 [26]. Most of the identified pri-miRNAs show a similar abundance across the different analysed tissues and irrespective of P supply (Figure 7). However, the abundance of miRNA156 is about 2-3-fold higher in the P-deficient cluster root tissues which is in agreement with reports identifying this miRNA as P-deficiency induced in white lupin and Arabidopsis [36]. Targets of miRNA156 include the SQUAMOSA PROMOTER BINDING PROTEIN-like family members SPL3/4/5. The pri-miRNA166 and 393 showed highest abundance in the MR and TR/ICR, respectively. This could either be a sign of higher expression in this tissue or of a higher turn-over followed by increased degradation of target mRNAs in the MCR and TCR.

## Discussion

### Metabolic acclimation to P-limitation

Cluster root formation is an important adaptive trait to cope with limiting P-availability in several plant species. Analysis of the molecular mechanisms underpinning this complex developmental process will give a better understanding of the ways plants cope with limited P supply. Advances in next-generation sequencing technology permit probing for transcriptome-wide changes in gene expression in non-model species such as white lupin. Incorporation of our RNA-seq data into the existing data set [26], thus generating an improved sequence resource, in conjunction with functional gene annotation, will allow white lupin to become a molecular model species for the analysis of cluster root development. We have started to exploit this resource by analysis of transcriptomic differences in several stages of cluster root formation. Phosphate starvation-induced changes in gene expression were very similar when comparing root tip and mature parts of P-sufficient with P-deficient plants, respectively. This included mainly well characterised genes involved in the uptake, re-distribution and homeostasis of phosphate, i.e. phosphate transporters, SPX-domain genes or genes involved in lipid remodelling. Very similar sets of genes have been identified in studies of the root transcriptome in species such as white lupin [26], Arabidopsis [37-39] and rice [40-42]. By contrast, tissue-specific differences identify functional specialisation of the different cluster root stages of P-starved plants. In the MCR changes in gene expression are geared to support the metabolic reactions involved in the synthesis and secretion of large amounts of organic acids, i.e. up-regulation of genes belonging to the TCA cycle or glycolysis. This confirms earlier results in a spatially higher resolution and on a transcriptome-wide scale [26,43]. Our results indicate that the root tip is not producing or exporting organic acids and depend for this on the mature part of the cluster root. However, the root tip increases Pi uptake and acquisition capacity to profit from the exudative burst and subsequently liberated phosphate from insoluble complexes.

Previously, a close link between the regulation of sulfate and nitrate homeostasis under phosphate deficiency has been shown [44-46]. We identify an extra level of complexity by which the uptake and translocation of these anions between the various tissues of the developing cluster root is regulated. In addition, sustained uptake of iron under phosphate starvation can lead to iron toxicity and therefore reduced iron uptake is a mechanism to protect meristem activity [47]. The down-regulation of a homolog of the transcriptional regulator of iron responses FER/FIT and a vacuolar iron transporter VIT in the TCR confirms this interconnection of iron and phosphate homeostasis and its importance for the root tip [40,48,49].

### Hormonal control of cluster root development

Cluster root formation provides insight into how plants use general regulatory components to form specialised structures to adapt to their environment. We show that white lupin uses the same set of plant hormones (i.e. auxin and cytokinin) and transcription factors identified in model plant species with a less complex root architecture, such as Arabidopsis and rice [50,51], to initiate and regulate cluster root formation. The increased expression of *CYC* and *CDK* genes towards the cluster root tip reflects the cell proliferation and divisions occurring in the meristematic zone of the tip and the initiation of laterals from pericycle cells in the differentiation zone [51]. Several genes of the auxin-mediated signalling cascade are differentially expressed in the analysed cluster root stages with higher transcript abundance in the root tip. The auxin efflux carriers of the PIN family are essential to generate the auxin gradient within the root by polar transport towards the tip following the ‘fountain model’, leading to the primed state of lateral root founder cells and thus controlling lateral root initiation [52,53]. Members of the LAX auxin-influx carrier family are involved in the auxin accumulation close to the lateral root primordium promoting lateral root emergence [54], while the Aux/IAA protein family in conjunction with auxin response factor (ARFs) regulate the expression of downstream genes [55]. The high expression of *YUC2* and *IAMT1* towards the cluster root tip points to the importance of local auxin synthesis in cluster root formation. YUC2 is directly involved in the synthesis of auxin and has been shown to be expressed in the root meristem in Arabidopsis [56,57]. This flavin-monooxygenase is part of the main auxin biosynthetic pathway and catalyses the conversion of the precursor indole-3-pyruvic acid into the active auxin IAA [57]. Recently, a role for root-derived auxin in cluster root formation has also been shown in white lupin [25] and here we identify YUC2 as a likely biosynthetic enzyme involved. *IAMT1* encodes an IAA carboxyl methyltransferases converting IAA to methyl-IAA ester and involved in the regulation of auxin homeostasis [58]. The two genes *NPH3* and *WAT1* regulate growth by modification of the subcellular targeting of the auxin efflux carrier PIN2 and secondary cell wall formation by altering root auxin concentration, respectively [59,60]. Recently, CK has also been identified as an important signalling component in the Pi-deficiency induced changes in lupin root architecture [26]. In addition, studies in Arabidopsis have shown that CK prevents the initiation of lateral roots in close proximity. The observed lower expression of the CK degrading enzyme CKX and the up-regulation of the CK receptor CRE1 towards the cluster root tip provides evidence that the formation of cluster roots depends on a localised accumulation and increased ability to perceive CK in this part of the root to allow for the dense formation of lateral roots [22].

### Differentially expressed transcription factors

Several transcription factors (TFs) known to regulate root meristem function and lateral root initiation in Arabidopsis are also differentially regulated in the three cluster root stages of white lupin. Members of the PLETHORA (PLT) family of TFs regulate cell proliferation, elongation and differentiation via an auxin-induced expression gradient along the root [61]. In Arabidopsis the GRAS TF family member SCARECROW (SCR), the zinc finger TF MAGPIE (MGP) and the HD-Zip TF WOX5 are major regulators in maintaining the stem cell niche in the quiescent centre of the root meristem. SCR is also involved in the differentiation process leading to the specific root tissue types [51]. Additionally, SCR forms a regulatory network together with the transcription factors PHABULOSA and PHAVOLUTA (PHB/PHV), two TFs also differentially expressed in the TCR and MCR, and microRNA165/6 to consolidate cell identities in the developing root [62]. Interestingly, PHB has recently been shown to regulate cytokinin (CK) signalling by activating the CK biosynthesis gene IPT7 [23]. Root hairs are an important site of nutrient uptake as they increase root surface area and in white lupin their formation is induced already two days after lateral root emergence [16]. In agreement with this is the expression of homologs of the central regulator of root hair formation RSL1 in the ICR and RSL2 towards the mature part of the cluster root. These bHLH-type transcription factors are essential for root hair development and consequently mutants of the corresponding genes in Arabidopsis, Lotus and Physcomitrella are deficient of root hairs [63-65].

The detected down-regulation of the lupin TF homolog of ERF6, a regulator of the response to oxidative stress [66], is in agreement with the ROS-related stress induction under phosphate limitation. The accumulation of H_2_O_2_ in the root cortex has been observed for P-deprived Arabidopsis plants [67]. Interestingly, we also found a contrasting expression of the TF UBP1 and peroxidases in the cluster root. In Arabidopsis UBP1 regulates the expression of peroxidases within the root tip, thereby modulating the distribution of ROS species and controlling the transition from proliferation to differentiation [32]. Together, these results may indicate that reactive oxygen species are signalling molecules, but also products of oxidative stress, that require a complex regulation of local concentrations in the developing white lupin cluster root.

### White lupin pri-miRNAs

In addition to plant hormones and transcription factors, miRNAs are also important components in the regulation of root development [68]. We have been able to identify several primary transcripts of miRNAs (pri-miRNAs) expressed in the white lupin cluster root. We could not identify a homolog of pri-miRNA399, a highly induced miRNA under P-deficiency, in our cluster root assembly. This is not surprising as the mature miRNA in white lupin has been found exclusively in leaves, showing an up-regulation under P-limited growth conditions [35], likely to serve as a phloem-mobile signal [69,70]. miRNA156 was more abundant in cluster roots than in the P-supplied roots. Although the function of this miRNA in the regulation of P homeostasis is not known, it is up-regulated under phosphate starvation in the roots of white lupin and Arabidopsis [35,36]. In Arabidopsis targets of miRNA166 are the TFs PHB and PHV, for which white lupin homologs show variation in transcript abundance across the cluster root tissues, and these form a regulatory circuit to control cell fate in the root meristem [62]. The targets of miRNA393 are several F-Box proteins of the TIR1 subfamily (TIR1, AFB1/2/3) of auxin receptors and a TF of the bHLH family (bHLH77). In agreement with this it has been shown that the modulation of auxin sensitivity under phosphate deficiency, responsible for the alteration in lateral root patterning, is dependent on TIR1 [19]. With auxin being the major signalling molecule controlling root development it is therefore likely that miRNA393 and TIR1 play a role in the auxin-mediated regulation of cluster root formation. For the other identified miRNAs transcript abundance was similar across the analysed root tissues. This is in agreement with findings that only a few pri-miRNA show an altered expression under abiotic stresses although the abundance of the corresponding mature miRNA changes. This discrepancy has been attributed to differential turn-over of the mature miRNA and the processing efficiency of the pri-miRNAs [71,72].

## Conclusions

In conclusion, cluster root formation is a complex process that integrates spatiotemporally developmental events from the sensing of nutrient limitation, especially phosphorus, to the formation of a morphologically highly organised structure. We have identified an intricate network of signalling components and accompanying changes in gene expression controlling the formation of cluster roots. This also allows the plant to acclimate to Pi limitation in the different developmental stages of the cluster root. A better understanding of such adaptive mechanisms will help in improving plants by increasing their phosphate acquisition efficiency.

## Methods

### Plant material

Plants were grown and tissue harvested identical to Florez-Sarasa *et al.*[73]. Briefly, seeds of white lupin (*Lupinus albus* L. cv. Kiev mutant) were sown and germinated in pots filled with washed sand. Seedlings were carefully removed and the roots gently washed free of sand. Seven-day-old seedlings of uniform size were then transferred to a solution culture system each holding 20 L of continuously aerated nutrient solution (pH5.5) with the following composition: 5 mM KNO_3_, 1.5 mM Ca(NO_3_)_2_, 1 mM MgSO_4_, 23.1 μM H_3_BO_3_, 0.38 μM ZnSO_4_, 0.29 μM Na_2_MoO_4,_ 0.16 μM CuSO_4,_ 4.85 μM MnSO_4_, 19.14 μM Fe-EDDHA and for P sufficient plants supplemented with 1 mM NH_4_H_2_PO_4_ for the + P treatment. The entire nutrient solution was fully changed twice a week and pH was checked daily and readjusted back to around pH5.5. The plants were grown in a temperature-controlled glasshouse, with tubs half-immersed in a basin maintained at 19–22°C. The tubs were randomized and rotated weekly. Roots at the 6 to 8-trifoliolate stage were excised as root tips (TR, 1.5 cm of the root apex) and mature root (MR, 1.5 cm from the root base) for P sufficient plants and root tip (TCR, 1.5 cm of the root apex, free of visible laterals), immature cluster root (ICR, 1 cm following the TCR with developing laterals) and mature cluster root (MCR, 1 cm with only mature laterals) for P-deficient plants identical to Florez-Sarasa *et al.*[73]. The experiment was performed in three independent biological replicates.

### RNA extraction and next generation sequencing

The total RNA from the roots tissues was extracted using the Spectrum Plant Total RNA kit (Sigma-Aldrich, Castle Hill, Australia) according to the manufacturer’s instructions. The integrity and quality of the total RNA was checked using NanoDrop 1000 Spectrophotometer and formaldehyde agarose gel electrophoresis. RNA was only used when the Abs260 nm/Abs280 nm ratio was >1.8.

For RNA-seq library synthesis, 1 μg of total RNA was first depleted of rRNA using the Ribo-Zero rRNA Magnetic Kit (Plant Seed/Root kit, Epicentre, Madison, USA). Sequencing libraries were generated using the TruSeq RNA Sample Prep Kit (Illumina, Scoresby, Australia). Sequencing was then performed on a Hiseq 1000 as a 2 × 101 bp paired-end run according to manufacturer’s instructions (Illumina, Scoresby, Australia). RNA-seq read data were deposited to the NCBI Sequence Read Archive (NCBI SRA) under accession number SRA145661.

### *De novo* transcriptome assembly and functional classification

For the assembly of RNA-seq reads into contigs the Velvet/Oases pipeline was applied [28]. In total 133,045,174 paired-end reads of the three replicates from the five tissues samples were obtained. These reads were of high quality with a Phred score of above +30 and a minimal length of 90 bases. For optimisation of Velvet assembly parameters a Perl-based script was used (VelvetOptimiser Version 2.2.5, Victorian Bioinformatics Consortium, Monash University) which identified an optimal k-mer length of 67 for the assembly. To combine the obtained contigs with LAGI01 [26], the two assemblies were merged using CD-HIT-EST [74]. For annotation, homology searches were performed with Blast2go [75] against the NCBI database using the blastx algorithm with a cut-off of E < 10^−15^and targeted searches using the BLAT algorithm [76] against the soybean (Gmax_189) and Arabidopsis (TAIR10) transcriptome releases. Finally, contigs below 200 bp in length, with no assigned annotation or identified as contamination were removed from the assembly to yield the Lupinus albus Gene Index Version 2 (LAGI02) with a total of 65,097 contigs (sequences supplied in Additional files 2 and 3). Because the highest similarity in homology searches was observed between the soybean and lupin transcriptomes, GO terms for LAGI02 contigs were assigned based on the Gmax_189 genome release.

### Gene expression and data analysis

For gene expression analysis RNA-seq reads obtained for the three biological replicates for each tissue were mapped to the LAGI02 transcriptome using the Bowtie tool [77]. For quantification of transcript abundances and identification of significant changes in transcript expression the Cuffdiff tool with a upper quartile normalisation and multi read correction was applied to obtain FPKM values [78]. Hierarchical clustering was performed using the Manhattan distance matrix of the heatmap.2 function in the gplots package of the R environment. Over-representation of GO terms within gene clusters was identified with the BiNGO plugin for Cytoscape using a hypergeometric test after Benjamini and Hochberg FDR correction with a significance level of p < 0.01 [79]. For predication of miRNA hairpin structures the Vienna RNA websuite was used [80].

### Availability of supporting data

All sequences for LAGI02 contigs and their annotation are available as additional files (Additional files 1, 2 and 3). RNA-seq read data has been deposited in the NCBI SRA database under accession SRA145672.

## Competing interests

The authors declare that they have no competing interests.

## Authors’ contributions

DS carried out the RNA-seq experiments, participated in the bioinformatic analyses and the drafting of the paper. HS and JW conceived of the study, participated in its design and helped to draft the manuscript. OB performed the bioinformatic analyses and drafted the manuscript. All authors read and approved the final manuscript.

## Supplementary Material

Additional file 1 Functional annotation of LAGI02 contigs. Click here for file

Additional file 2 LAGI02 sequences in fasta format. Click here for file

Additional file 3 LAGI02 sequences in fasta format. Click here for file

Additional file 4 **LAGI02 (Lupinus albus Gene Index version 02) characteristics. A)** Length distribution of LAGI02 contigs. In total LAGI02 contains 65,097 contigs above a length of 200 bp with a total length of 105,789,289 bp and an average length of 1,625 bp. **B)** Species distribution of BLAST hits for LAGI02 contigs. More than 87% of primary hits are against other legume species (cut-off E < 10E-15). Click here for file

Additional file 5 **Classification of LAGI02 contigs to gene ontology (GO) terms.** LAGI02 contigs were assigned to plant GOslim terms within each of the three main ontologies biological process, cellular component and molecular function. For comparison the distribution of GOslim terms for LAGI02 and the soybean (genome release Gmax_189) classification are shown. Numbers give percentages of each GOslim term within main ontologies. Click here for file

Additional file 6 **MapMan bin classification of LAGI02 contigs.** LAGI02 contigs were assigned to MapMan bins using the Mercator pipeline (http://mapman.gabipd.org). Shown is a comparison of MapMan bin classifications of the LAGI02 annotation with those for soybean and Arabidopsis based on the Gmax_189 and TAIR10 genome releases, respectively, downloaded from the MapMan website. Click here for file

Additional file 7 FPKM values after expression quantification of LAGI02 contigs. Click here for file

Additional file 8 **Exemplary read visualisation for LAGI02 contigs differentially expressed across tissues.** Three examples of genes differentially expressed across tissues. Shown are read mappings to the LAGI02 transcriptome assembly visualised using the IGV browser. MLS has high read numbers in the MCR and ICR, PAP10 in all P-deficient tissues (MCR, ICR, TCR) and PHO2 shows higher transcript abundance in the P-sufficient tissues (MR, TR), likely because of miRNA399 induced degradation in the P-deficient tissues. Abbreviations for tissue samples: +P) MR, mature root; TR: tip of root; −P) MCR, mature cluster root; ICR, immature cluster root; TCR, tip of cluster root Abbreviations for genes: MLS, MALATE SYNTHASE; PAP10, PURPLE ACID PHOSPHATASE10; PHO2, PHOSPHATE2. Click here for file

Additional file 9 **MapMan visualisation of differentially gene expression in root tips and mature parts of P-sufficient and -deficient roots.** Known P starvation responsive genes show a similar differential expression in comparisons of the mature root tissues and root tips of plants grown under -P and + P conditions, i.e. MCR vs. MR and TCR vs. TR. Shown are P-transporters, genes involved in phosphate uptake, in the regulation of P homeostasis and genes related to metabolic reactions in the acclimation to P limitation. Shown are log2 values for FPKM ratios. Abbreviations: PHT, PHOSPHATE TRANSPORTER; SPX, SPX-domain containing; TF, transcription factors; PHO1, PHOSPHATE1; PAP, PURPLE ACID PHOSPHATASE. Click here for file

Additional file 10 **Differential expression of genes in the mature part and the root tip of the cluster root involved in membrane transport.** MapMan visualisation of differential expression for genes involved in the transport of ions and other solutes across various cellular membranes. Shown are log_2_ values for the FPKM ratios of MCR vs. TCR. Click here for file

Additional file 11 Enriched GO terms in Clusters 1 and Clusters 2/3 for contigs differentially expressed across cluster root tissues. Click here for file

Additional file 12 **Secondary structures for white lupin and soybean pre-miRNA hairpins.** Shown are the hairpin structures of white lupin pre-miRNA and their corresponding homologs in soybean with sequences of mature miRNAs in red. Hairpin structures were predicted using the Vienna websuite (http://rna.tbi.univie.ac.at/) and compared to predictions for homologs in miRBase (http://www.mirbase.org/). Click here for file
