# Supplementary figures and images for: RNA-seq analysis identifies an intricate regulatory network controlling cluster root development in white lupin
Source: BMC Genomics. 2014 Mar 25;15:230. doi: 10.1186/1471-2164-15-230 (PMC4028058; doi:10.1186/1471-2164-15-230)

**A**

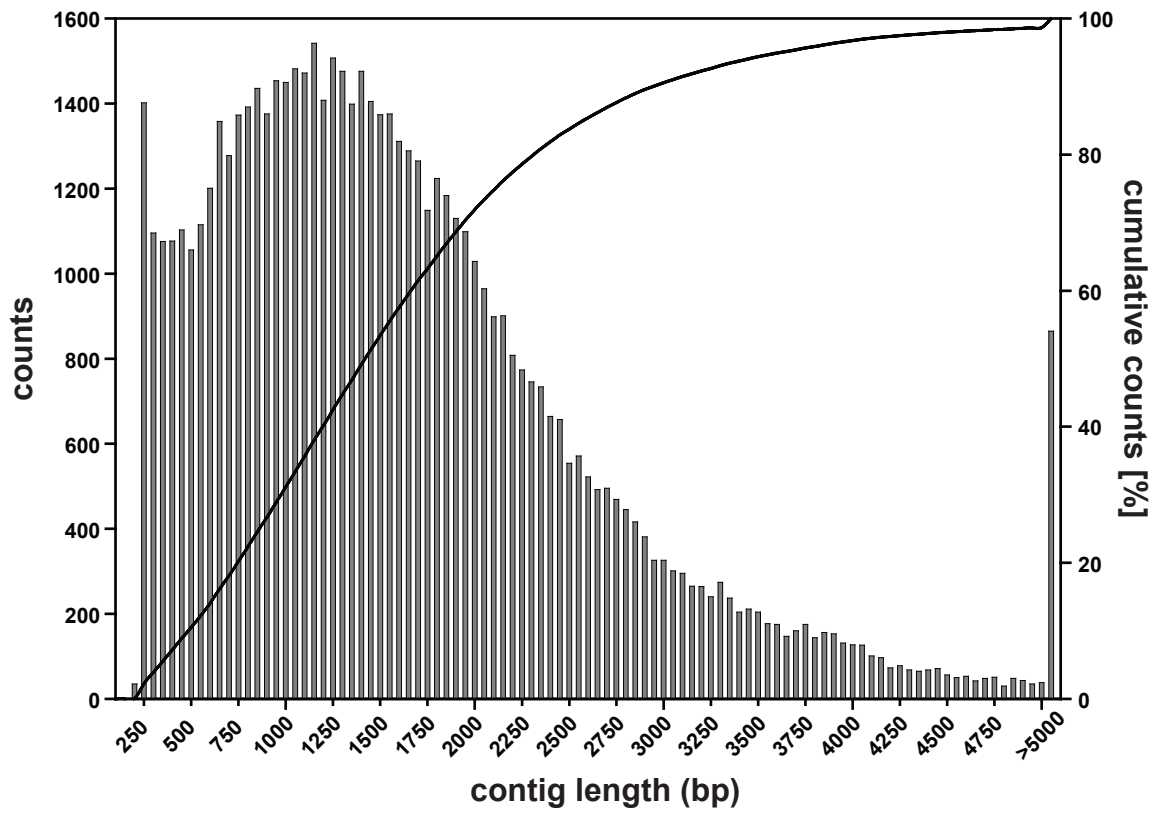

**B**

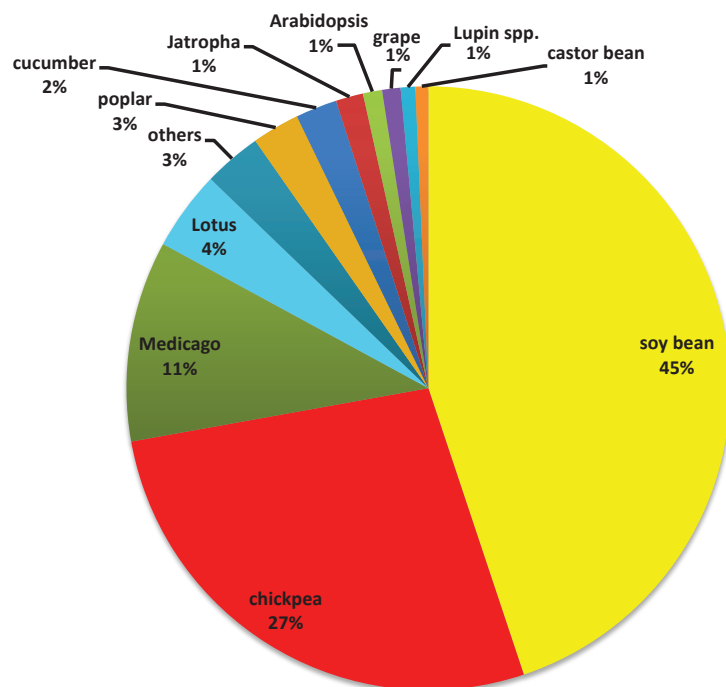

Supplement: Additional file 4 — LAGI02 (Lupinus albus Gene Index version 02) characteristics. A) Length distribution of LAGI02 contigs. In total LAGI02 contains 65,097 contigs above a length of 200 bp with a total length of 105,789,289 bp and an average length of 1,625 bp. B) Species distribution of BLAST hits for LAGI02 contigs. More than 87% of primary hits are against other legume species (cut-off E < 10E-15). [file 1471-2164-15-230-S4.pdf]

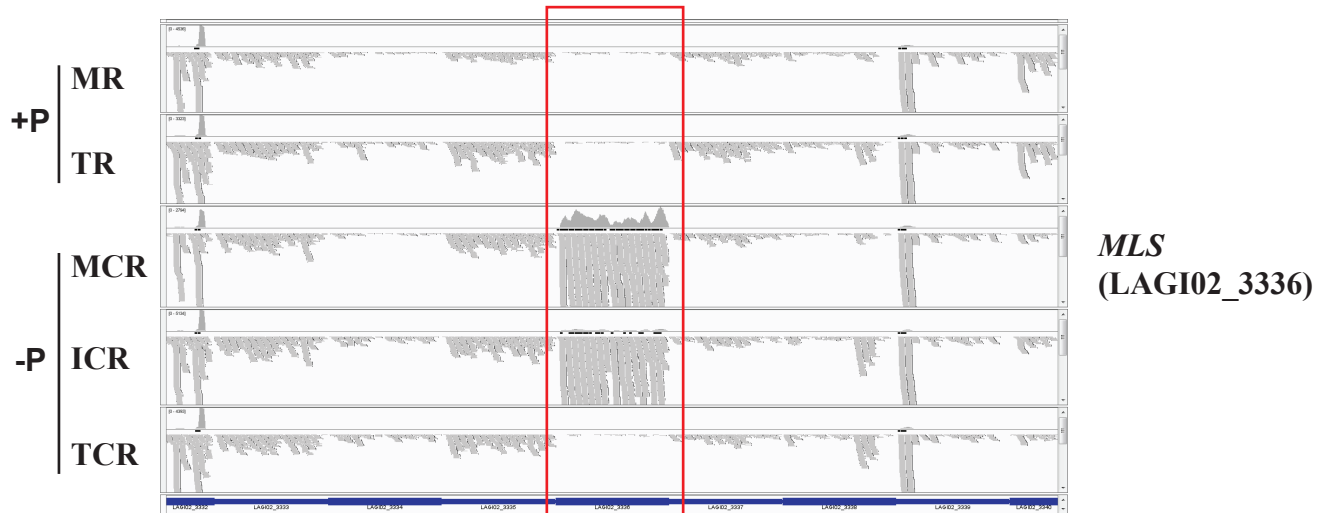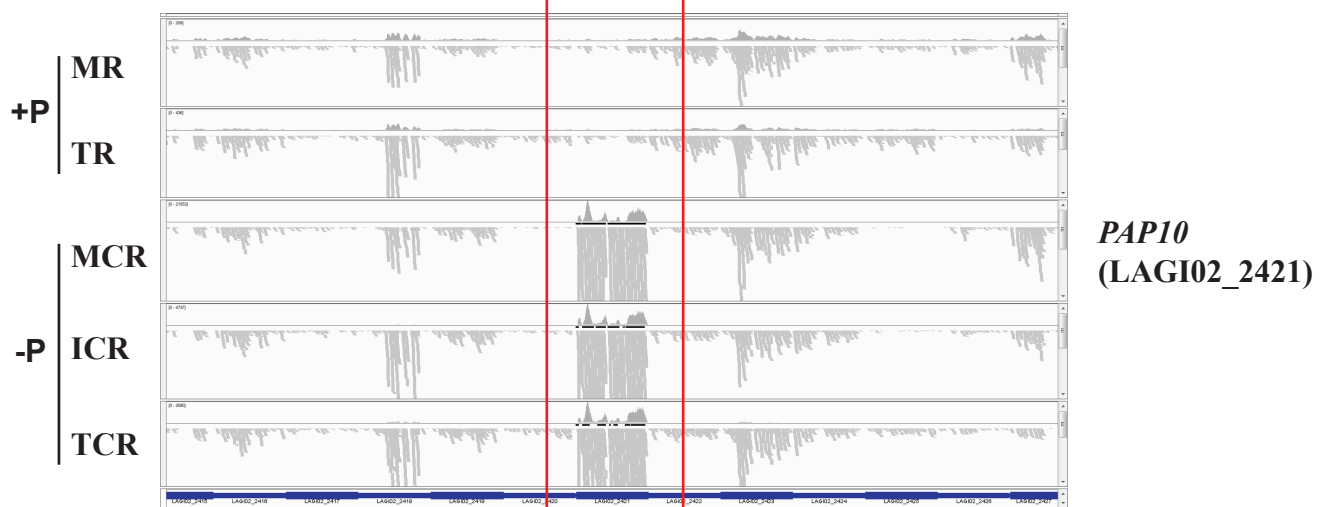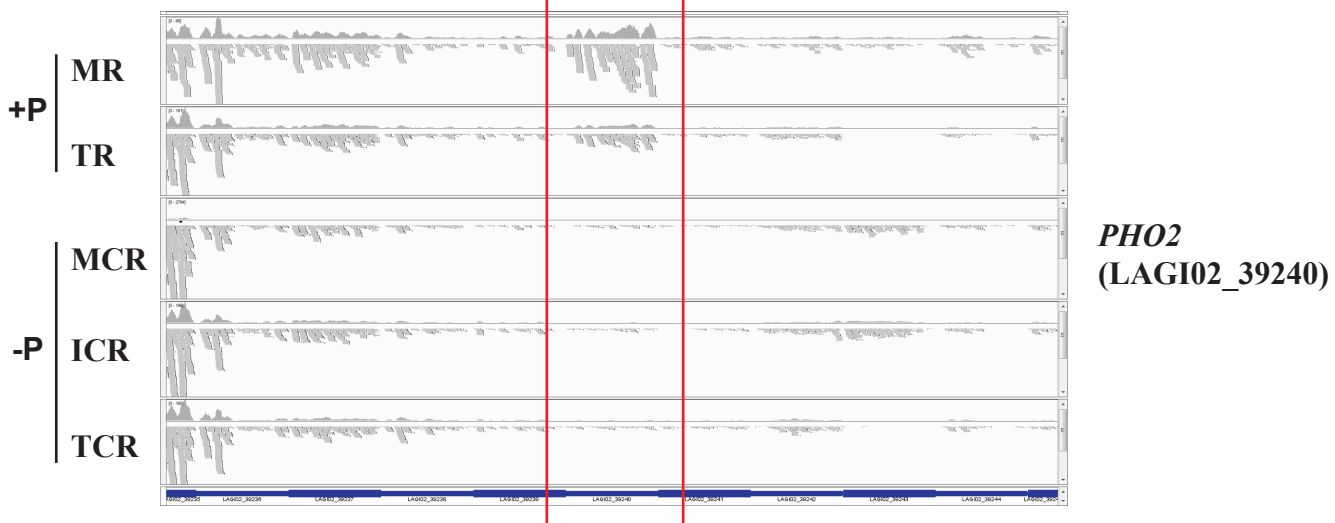

Supplement: Additional file 8 — Exemplary read visualisation for LAGI02 contigs differentially expressed across tissues. Three examples of genes differentially expressed across tissues. Shown are read mappings to the LAGI02 transcriptome assembly visualised using the IGV browser. MLS has high read numbers in the MCR and ICR, PAP10 in all P-deficient tissues (MCR, ICR, TCR) and PHO2 shows higher transcript abundance in the P-sufficient tissues (MR, TR), likely because of miRNA399 induced degradation in the P-deficient tissues. Abbreviations for tissue samples: +P) MR, mature root; TR: tip of root; −P) MCR, mature cluster root; ICR, immature cluster root; TCR, tip of cluster root Abbreviations for genes: MLS, MALATE SYNTHASE; PAP10, PURPLE ACID PHOSPHATASE10; PHO2, PHOSPHATE2. [file 1471-2164-15-230-S8.pdf]

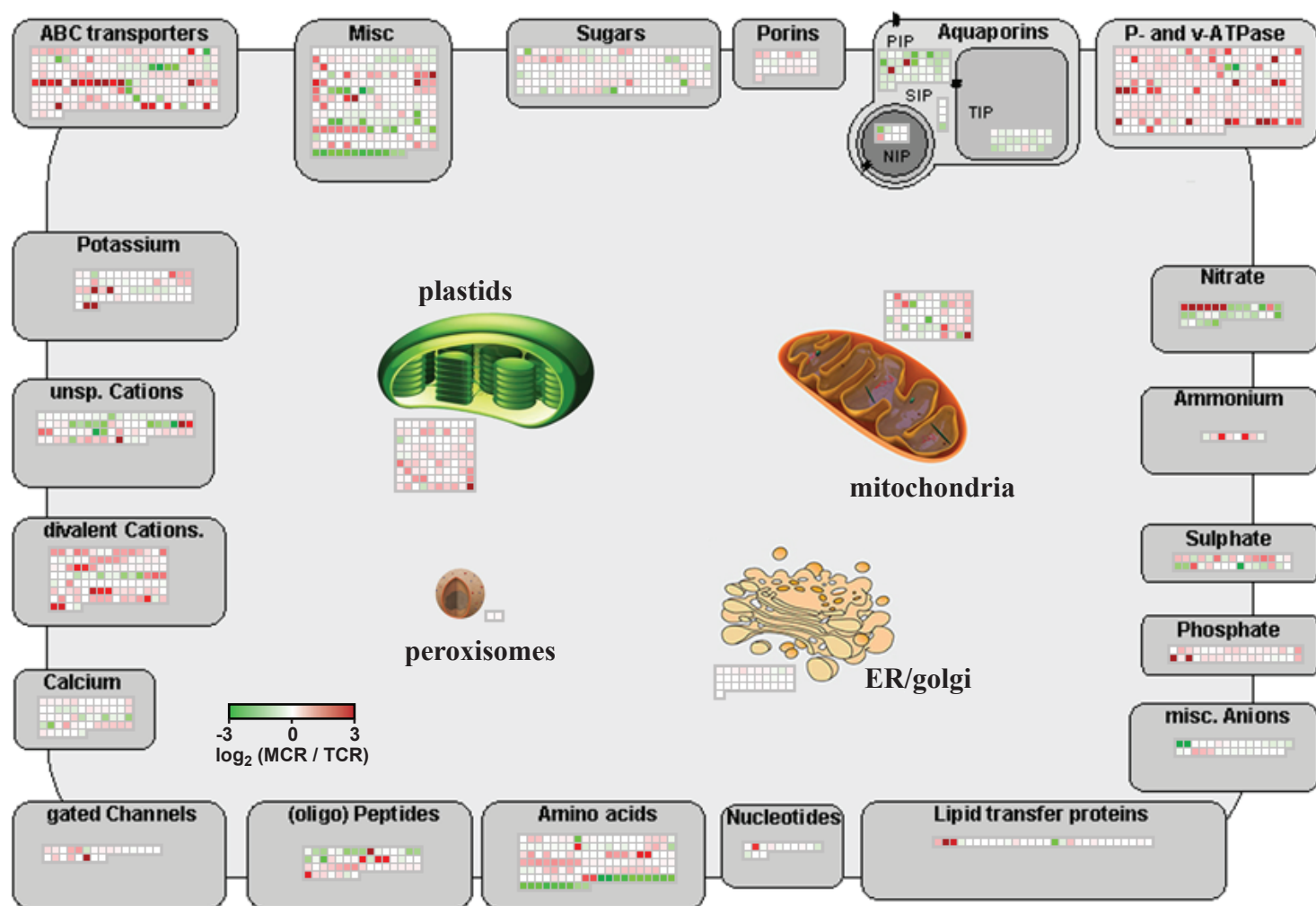

Supplement: Additional file 10 — Differential expression of genes in the mature part and the root tip of the cluster root involved in membrane transport. MapMan visualisation of differential expression for genes involved in the transport of ions and other solutes across various cellular membranes. Shown are log2 values for the FPKM ratios of MCR vs. TCR. [file 1471-2164-15-230-S10.pdf]
