# Supplementary material for: RNA-seq analysis identifies an intricate regulatory network controlling cluster root development in white lupin
Source: BMC Genomics. 2014 Mar 25;15:230. doi: 10.1186/1471-2164-15-230 (PMC4028058; doi:10.1186/1471-2164-15-230)
Supplement: Additional file 5 — Classification of LAGI02 contigs to gene ontology (GO) terms. LAGI02 contigs were assigned to plant GOslim terms within each of the three main ontologies biological process, cellular component and molecular function. For comparison the distribution of GOslim terms for LAGI02 and the soybean (genome release Gmax_189) classification are shown. Numbers give percentages of each GOslim term within main ontologies. [file 1471-2164-15-230-S5.pdf]

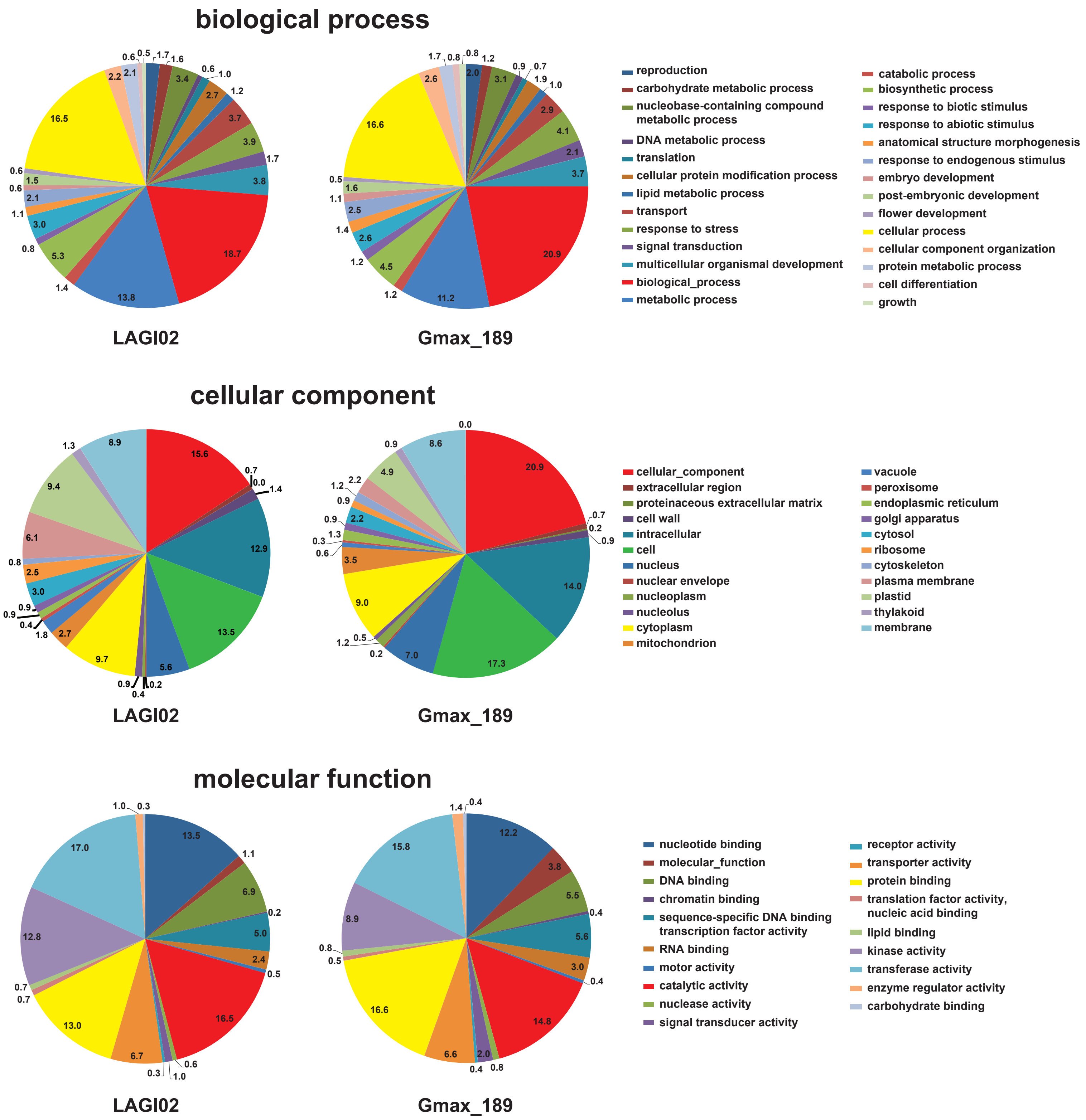

biological process
LAGI02
Gmax_189
reproduction
carbohydrate metabolic process
nucleobase-containing compound metabolic process
DNA metabolic process
translation
cellular protein modification process
lipid metabolic process
transport
response to stress
signal transduction
multicellular organismal development
biological_process
metabolic process
catabolic process
biosynthetic process
response to biotic stimulus
response to abiotic stimulus
anatomical structure morphogenesis
response to endogenous stimulus
embryo development
post-embryonic development
flower development
cellular process
cellular component organization
protein metabolic process
cell differentiation
growth
cellular component
LAGI02
Gmax_189
cellular_component
extracellular region
proteinaceous extracellular matrix
cell wall
intracellular
cell
nucleus
nuclear envelope
nucleoplasm
nucleolus
cytoplasm
mitochondrion
vacuole
peroxisome
endoplasmic reticulum
golgi apparatus
cytosol
ribosome
cytoskeleton
plasma membrane
plastid
thylakoid
membrane
molecular function
LAGI02
Gmax_189
nucleotide binding
molecular_function
DNA binding
chromatin binding
sequence-specific DNA binding transcription factor activity
RNA binding
motor activity
catalytic activity
nuclease activity
signal transducer activity
receptor activity
transporter activity
protein binding
translation factor activity, nucleic acid binding
lipid binding
kinase activity
transferase activity
enzyme regulator activity
carbohydrate binding
