# Supplementary material for: RNA-seq analysis identifies an intricate regulatory network controlling cluster root development in white lupin
Source: BMC Genomics. 2014 Mar 25;15:230. doi: 10.1186/1471-2164-15-230 (PMC4028058; doi:10.1186/1471-2164-15-230)
Supplement: Additional file 6 — MapMan bin classification of LAGI02 contigs. LAGI02 contigs were assigned to MapMan bins using the Mercator pipeline (http://mapman.gabipd.org). Shown is a comparison of MapMan bin classifications of the LAGI02 annotation with those for soybean and Arabidopsis based on the Gmax_189 and TAIR10 genome releases, respectively, downloaded from the MapMan website. [file 1471-2164-15-230-S6.pdf]

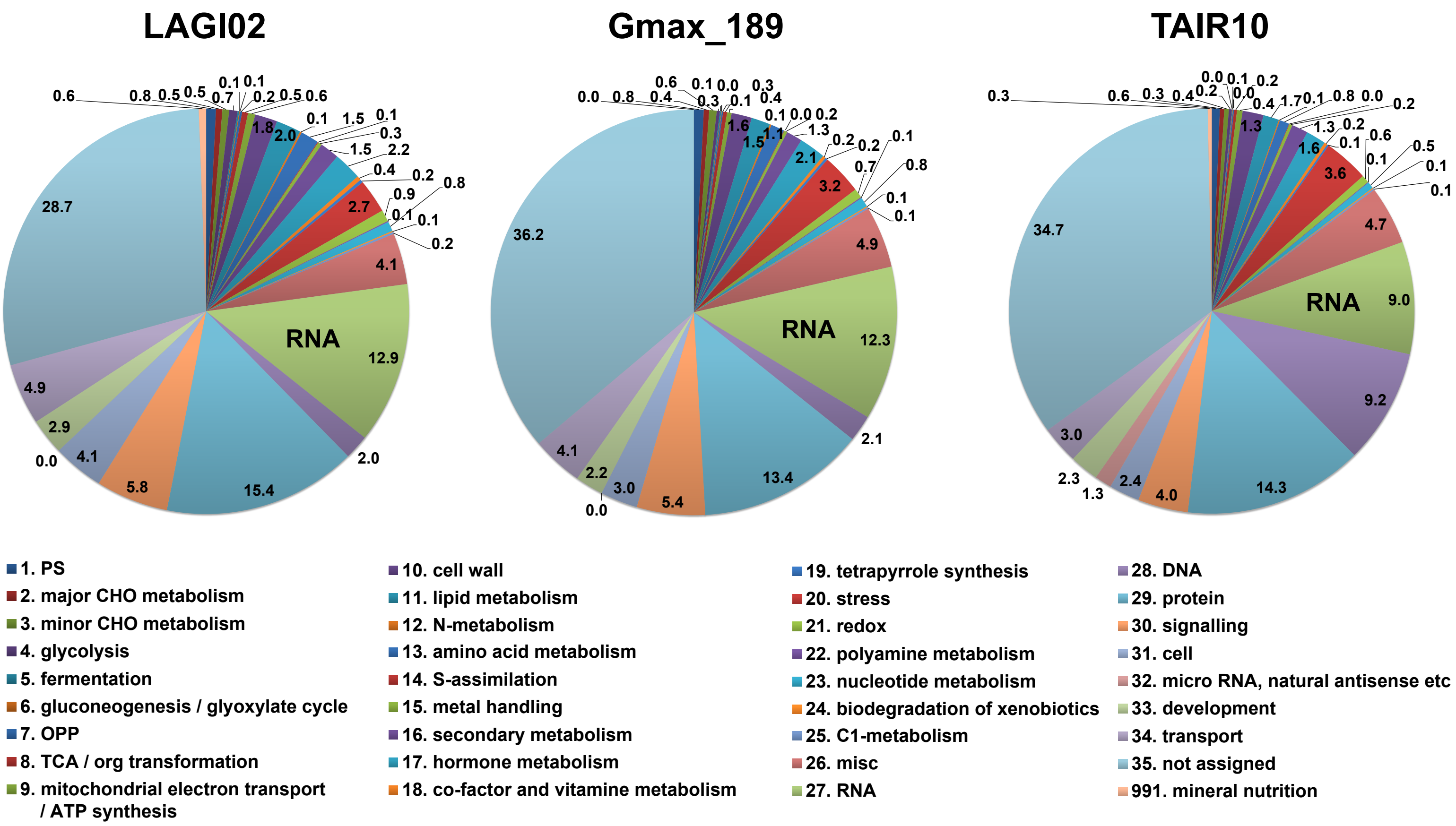
LAGI02
RNA
Gmax_189
RNA
TAIR10
RNA
1. PS
2. major CHO metabolism
3. minor CHO metabolism
4. glycolysis
5. fermentation
6. gluconeogenesis / glyoxylate cycle
7. OPP
8. TCA / org transformation
9. mitochondrial electron transport / ATP synthesis
10. cell wall
11. lipid metabolism
12. N-metabolism
13. amino acid metabolism
14. S-assimilation
15. metal handling
16. secondary metabolism
17. hormone metabolism
18. co-factor and vitamine metabolism
19. tetrapyrrole synthesis
20. stress
21. redox
22. polyamine metabolism
23. nucleotide metabolism
24. biodegradation of xenobiotics
25. C1-metabolism
26. misc
27. RNA
28. DNA
29. protein
30. signalling
31. cell
32. micro RNA, natural antisense etc
33. development
34. transport
35. not assigned
991. mineral nutrition
