# Supplementary material for: RNA-seq analysis identifies an intricate regulatory network controlling cluster root development in white lupin
Source: BMC Genomics. 2014 Mar 25;15:230. doi: 10.1186/1471-2164-15-230 (PMC4028058; doi:10.1186/1471-2164-15-230)
Supplement: Additional file 9 — MapMan visualisation of differentially gene expression in root tips and mature parts of P-sufficient and -deficient roots. Known P starvation responsive genes show a similar differential expression in comparisons of the mature root tissues and root tips of plants grown under -P and + P conditions, i.e. MCR vs. MR and TCR vs. TR. Shown are P-transporters, genes involved in phosphate uptake, in the regulation of P homeostasis and genes related to metabolic reactions in the acclimation to P limitation. Shown are log2 values for FPKM ratios. Abbreviations: PHT, PHOSPHATE TRANSPORTER; SPX, SPX-domain containing; TF, transcription factors; PHO1, PHOSPHATE1; PAP, PURPLE ACID PHOSPHATASE. [file 1471-2164-15-230-S9.pdf]

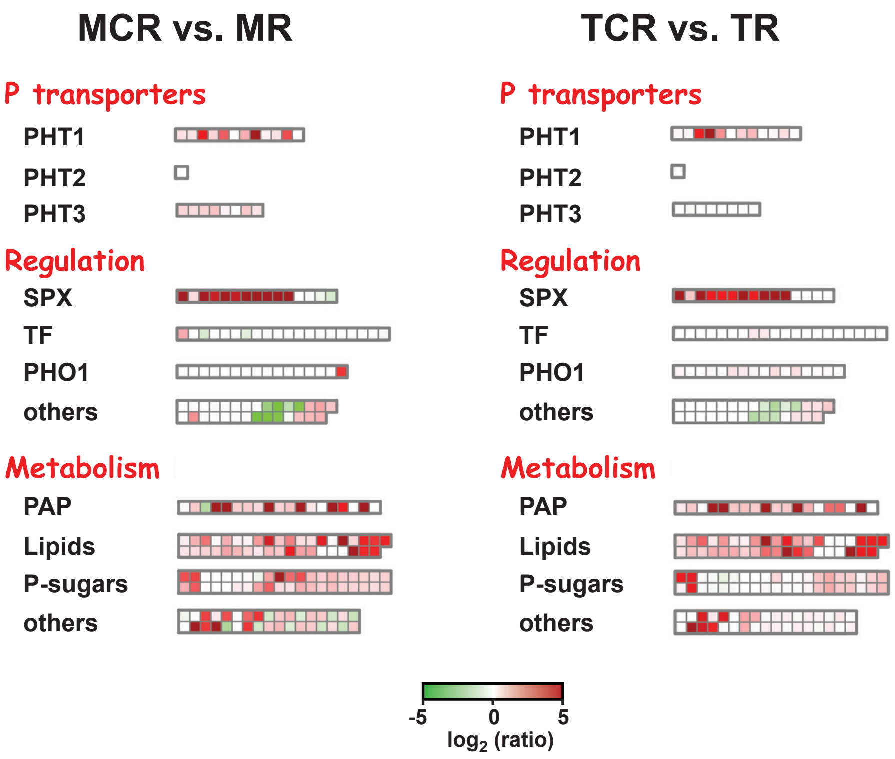

MCR vs. MR
TCR vs. TR
P transporters
PHT1
PHT2
PHT3
Regulation
SPX
TF
PHO1
others
Metabolism
PAP
Lipids
P-sugars
others
-5
0
5
log2 (ratio)
