# Supplementary material for: RNA-seq analysis identifies an intricate regulatory network controlling cluster root development in white lupin
Source: BMC Genomics. 2014 Mar 25;15:230. doi: 10.1186/1471-2164-15-230 (PMC4028058; doi:10.1186/1471-2164-15-230)
Supplement: Additional file 12 — Secondary structures for white lupin and soybean pre-miRNA hairpins. Shown are the hairpin structures of white lupin pre-miRNA and their corresponding homologs in soybean with sequences of mature miRNAs in red. Hairpin structures were predicted using the Vienna websuite (http://rna.tbi.univie.ac.at/) and compared to predictions for homologs in miRBase (http://www.mirbase.org/). [file 1471-2164-15-230-S12.pdf]

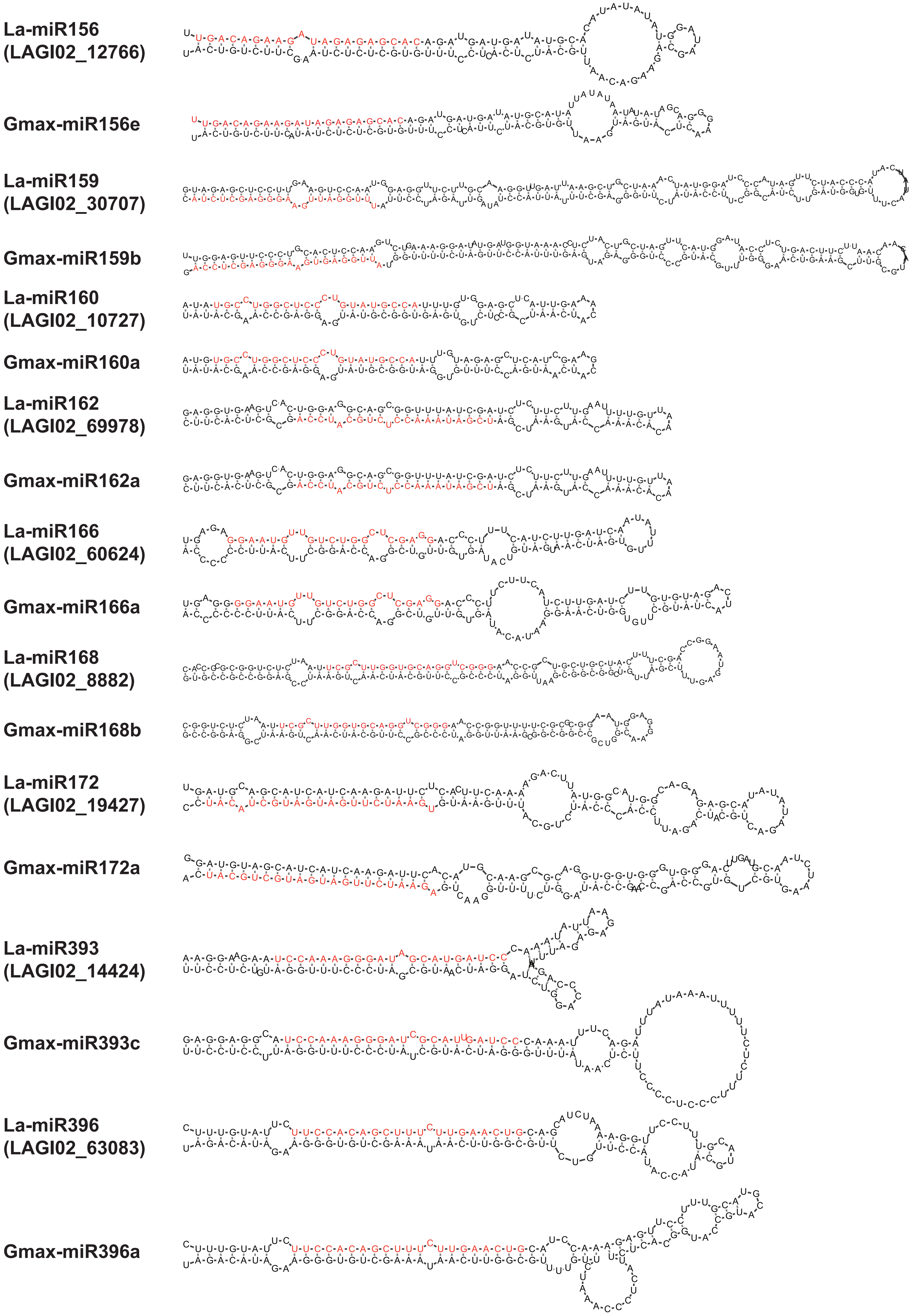
La-miR156
(LAGI02_12766)
Gmax-miR156e
La-miR159
(LAGI02_30707)
Gmax-miR159b
La-miR160
(LAGI02_10727)
Gmax-miR160a
La-miR162
(LAGI02_69978)
Gmax-miR162a
La-miR166
(LAGI02_60624)
Gmax-miR166a
La-miR168
(LAGI02_8882)
Gmax-miR168b
La-miR172
(LAGI02_19427)
Gmax-miR172a
La-miR393
(LAGI02_14424)
Gmax-miR393c
La-miR396
(LAGI02_63083)
Gmax-miR396a
